# The effect of shear-dependent flocculation on the multimodality of effective particle size distributions in a gravel-bed river during high flows

**DOI:** 10.1007/s11368-023-03455-5

**Published:** 2023-03-17

**Authors:** R. Maltauro, M. Stone, A. L. Collins, B. G. Krishnappan, U. Silins

**Affiliations:** 1 Department of Geography & Environmental Management, University of Waterloo, Waterloo, ON Canada; 2 Net Zero and Resilient Farming, Rothamsted Research, Okehampton, Devon UK; 3 Environment Canada, Burlington, ON Canada; 4 Department of Renewable Resources, University of Alberta, Edmonton, AB Canada

**Keywords:** Suspended particulate matter, Microflocs, Macroflocs, Shear stress, LISST 200X

## Abstract

**Purpose:**

Multimodal effective particle size distributions (EPSDs) develop as flocculation and particle breakage occur dynamically in a fluid shear and such distributions have been previously reported in coastal and estuarine waters to understand flocculation processes. Here, we use time varying multimodal EPSDs and hydraulic parameters (discharge and bed shear stress) to assess freshwater flocculation in a gravel-bed river in southern Alberta, Canada.

**Methods:**

Instantaneous discharge, volume concentration (VC), and EPSD of suspended solids were measured during three high discharge events at four study sites in a 10 km reach of the Crowsnest River. The EPSD and VC of suspended solids (< 500 µm) were measured in the centroid of flow with a LISST-200x. Bed shear stress for measured discharge was obtained using a flow model, MOBED.

**Results:**

Multimodal EPSDs consisted of primary particles, flocculi, microflocs, and macroflocs. Shear dependent flocculation was consistently observed for all sites and events, due to low and high shear stress flocculation, particle breakage, and mobilization of tributary sub-catchment derived particles. Higher shear stress limited flocculation to smaller floc sizes, while lower bed shear stress conditions created higher volumes of macroflocs.

**Conclusion:**

Flocculation and particle breakage processes based on relationships between particle size and hydraulic properties presented herein have implications for advancing fine sediment transport models by a variable cohesion factor as a function of floc size class.

## Introduction

 “Excess” suspended particulate matter (SPM) in rivers is a key driver of water quality degradation (Wood and Armitage 1997; Brunke 1999; Bilotta and Brazier 2008). SPM is an important pollutant vector (Stone and Mudroch 1989; Walling et al. 2003; Collins et al. 2005) that can affect aquatic ecosystem health (Kemp et al. 2011; Jones et al. 2012; Wilkes et al. 2019) and challenge water treatability and the provision of safe drinking water (Emelko et al. 2011; Bladon et al. 2014). Early research on fluvial SPM assumed that particulate matter was primarily transported as individual particles and interaction with the channel bed was limited (Einstein et al. 1940; Krishnappan 2007; Walling and Collins 2016). However, it is now more commonly accepted that cohesive solids (< 63 µm) are mainly transported as flocculated particles due to the geochemical and related electrochemical surface properties of these materials (Lick 1982; Lick et al. 1992; Krishnappan 2007; Williams et al. 2008; Droppo and Krishnappan 2016; Lai et al. 2018). These aggregated particles formed in the water column, known as flocs, typically consist of a complex mixture of inorganic (e.g., clays and silts) and organic (e.g., extracellular polymeric substances – EPS) particles as well as microbial organisms (Droppo 2001; Ho et al. 2022). Further, soil aggregates formed on the land surface can eventually reach the water column and be quickly incorporated to SPM, forming hybrid soil aggregate-floc particles (Droppo et al. 2005b). Floc composition can influence the morphology (e.g., floc size) and transport properties of aggregated particles, and both these attributes have been observed to have high spatial and temporal variability in rivers (Petticrew 2005; Phillips and Walling 2005).

The need for improved knowledge of flocculation and SPM transport processes in gravel-bed rivers draining forested mountainous landscapes has emerged as a critical research area. These rivers often drain forested landscapes that are critical for source water supply (Emelko et al. 2011, 2016; Stone et al. 2021), regional biodiversity, and landscape scale ecological integrity (Hauer et al. 2016). However, there is increasing concern regarding anthropogenic and natural landscape disturbances such as harvesting, wildfire, municipal wastewater discharge, agriculture,and drought (Schindler and Donahue 2006; Flannigan et al. 2009; Emelko et al. 2011; Watt et al. 2021) which increases the delivery of “excess” fine particulate matter from hillslopes to stream networks. The boundary shear stress conditions in gravel-bed rivers influence fine sediment transport dynamics via intra-gravel infiltration and exfiltration mechanisms (Casas-Mulet et al. 2017). Such mechanisms permit the temporary storage of fine sediment in gravel-beds, which can influence nutrient and contaminant fluxes (Walling and Collins 2016) and salmonid spawning habitats (Sear et al. 2014; Collins et al. 2014). Despite the widely reported deleterious impacts of “excess” SPM and even more recent advances in understanding the role of natural flocculation processes on these impacts, the factors controlling flocculation and its effects on the transport and fate of SPM in gravel-bed river systems still require further study (Mikkelsen et al. 2006; Krishnappan 2022). Such information is required to refine the flocculation component of fine sediment transport models in these river systems (Petticrew 2005; Koiter et al. 2015; Stone et al. 2021).

Flocs are created under different conditions of fluid shear by the dynamic interaction of particle types such as primary particles, flocculi, microflocs, and macroflocs (Lee et al. 2012). Primary particles can aggregate into tightly packed flocs referred to as flocculi (Lee et al. 2012). Under favorable biophysical conditions, these particles combine with other flocculi to form microflocs that can grow into more loosely-bound macroflocs (Eisma 1986; Mikkelsen et al. 2006; Lee et al. 2012; Ho et al. 2022). Flocculi are formed by strong Coulombic attraction between negatively charged clay surfaces and sporadic positive sites at the edge of these minerals (face-to-edge attachment) and these particles seldom disaggregate into primary particles (Lee et al. 2012). In contrast, micro and macrofloc assemblages form due the presence of extracellular polymeric substances (EPS), which can lower the resistance of flocs to breakage (Droppo 2001; Ho et al. 2022). In a fluid shear, flocculation and particle breakage coexists interactively, causing particle size distributions (PSDs) to be dynamic (Gibbs et al. 1989; Phillips and Walling 1999; Le et al. 2020). Accordingly, PSDs are often multimodal, presenting multiple modal peaks (Stone and Krishnappan 2003; Mikkelsen et al. 2006; Lee et al. 2012), and lognormal, with skewness depending upon the dominant size classes in suspension (Blott and Pye 2001; Lee et al. 2012).

Floc size is a critical parameter controlling the transport and fate of SPM. The hierarchical assemblage from primary particles to macroflocs changes particle size, shape, structure, and density (Ho et al. 2022), ultimately affecting the deposition and the downstream propagation of suspended particles (Droppo 2001; Krishnappan 2007; Maerz et al. 2011). Despite extensive research on flocculation in both engineered and natural systems (Droppo et al. 2005a), advancing knowledge of flocculation in environments undergoing cumulative development pressures is required to refine fine sediment transport models (Krishnappan 1991; Stone et al. 2021). Time-varying multimodal PSD data have been used previously to understand possible controls and to investigate particle and aggregate dynamics in coastal and estuarine waters (Gibbs et al. 1989; Mikkelsen et al. 2006; Lee et al. 2012; Le et al. 2020). Here, we adopt this approach to evaluate multimodality in the EPSDs of SPM in a gravel-bed river under varying conditions of fluid shear and SPM concentrations at a range of spatial and temporal scales. The specific objectives of this study were to (1) evaluate changes in discrete groups of particles and aggregates (primary particles, flocculi, microflocs and macroflocs) as a function of SPM volume concentration (VC), discharge (Q) and bed shear stress (τ) during spring freshet and stormflow, and; (2) characterize the spatial and temporal variability in EPSD multimodality caused by the mixing of multiple particle and aggregate size groups under flocculation and erosion/resuspension. The investigation of spatial and temporal EPSD multimodality, in situ, is critical for the refinement of SPM transport models, which can ultimately benefit watershed management by improving estimates of the transport and fate of sediment and associated contaminant in downstream aquatic environments.

## Materials and methods

### Site description

The Crowsnest River drains an area of ~ 679 km^2^ on the eastern slopes of the Rocky Mountains in southwestern Alberta. The headwaters of this river originate in the upper montane snowmelt-dominated regions that drain into Crowsnest Lake (1357 m.a.s.l.). The river flows through the Municipality of Crowsnest Pass and then into the Oldman Reservoir (1113 m.a.s.l.) (Watt et al. 2021). Average annual precipitation ranges from ~ 400 to 1000 mm year^−1^ and ~ 30% is snow fall (Alberta Agriculture, Forestry and Rural Economic Development and Alberta Climate Information Service (ACIS) 2021). Streamflow in the Crowsnest River is strongly influenced by snowmelt, which occurs between the late spring and early summer seasons, typically peaking in early June (Waterline 2013). Peak flows occur due to rain-on-snow events, or in response to large convective or frontal storms in the summer (Stone et al. 2014). Baseflow in the Crowsnest River, however, is dominated by groundwater inputs from alluvial aquifers in the river valley (Waterline 2013). Regional geology consists of limestone, dolomite, shales, mudstones, and fine-grained sandstone, while surficial geology comprises thin colluvium, fine-grained till blankets and till veneers (Silins et al. 2014; Stone et al. 2014). Glacial deposits in the study basin are potential sources of fine-grained materials that enter river channels either through hillslope surface or channel bank erosion (Silins et al. 2014; Stone et al. 2014).

### Sampling program

**Fig. 1:**
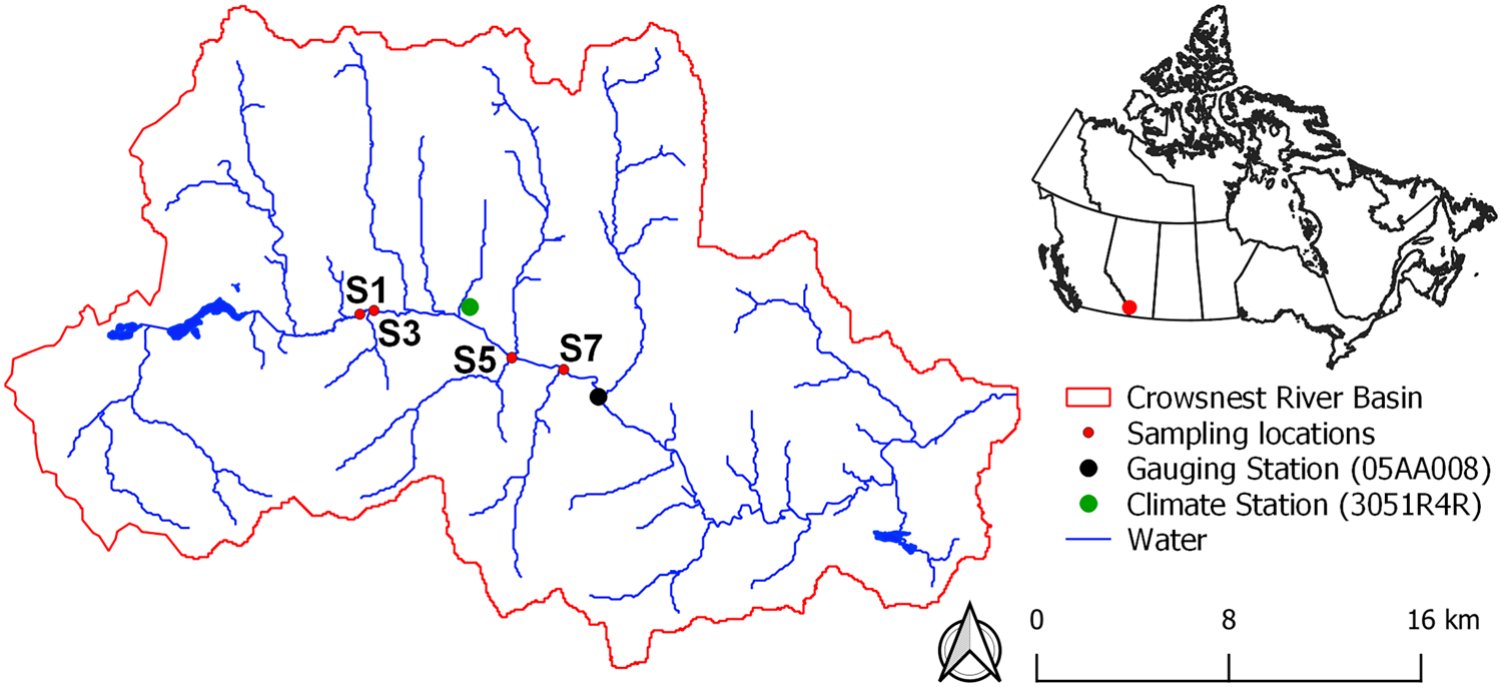
Location of study sites in the Crowsnest River basin

**Fig. 2:**
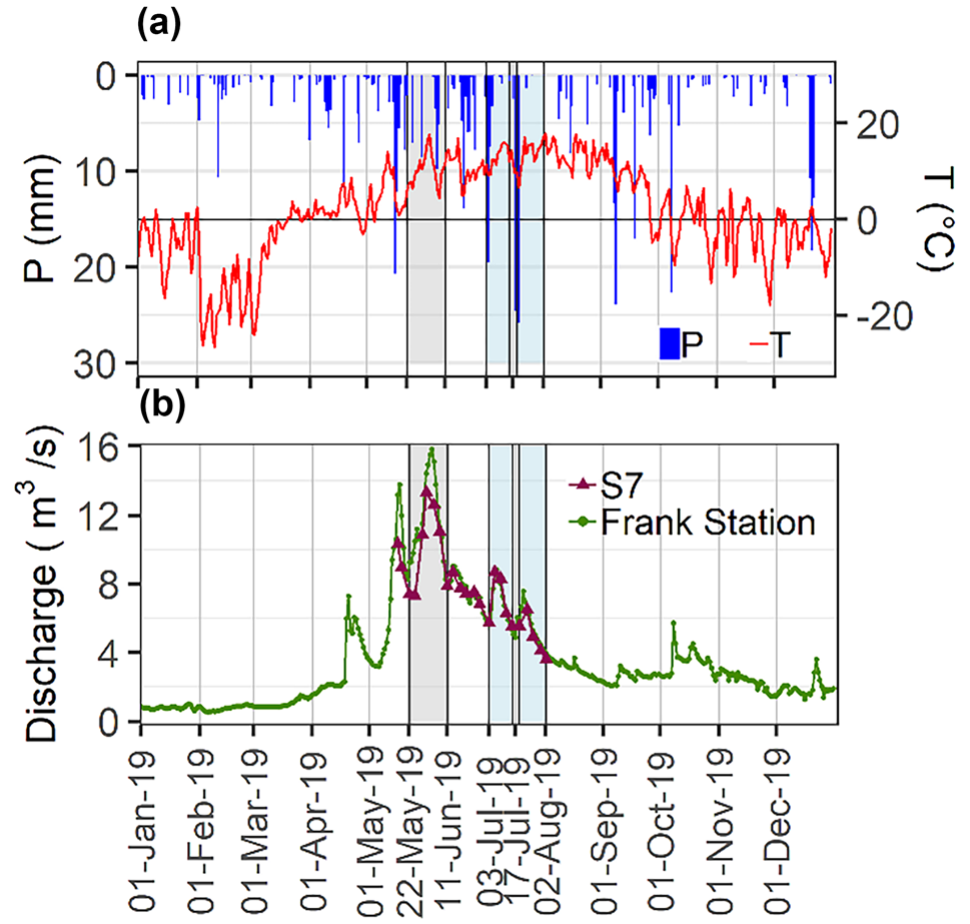
**a** Daily mean precipitation and temperature at the Crowsnest station (3051R4R) (Alberta Agriculture, Forestry and Rural Economic Development and Alberta Climate Information Service (ACIS) 2021); **b** daily mean values of discharge at the Crowsnest River – Frank (05AA008) Gauging Station (Environment Canada 2021), and instantaneous discharge measurements at S7 during the study period (burgundy line). Shaded areas in gray represent event 1, and those in blue events 2 and 3

The study was conducted at four locations along a 10 km reach of the Crowsnest River (Fig. 1) that represent a continuum of cumulative impacts from both natural and anthropogenic disturbances in the Crowsnest River Basin. Land disturbance types in the watershed include agriculture, industry, mining, municipal development and wildfire and these pressures cumulatively increase downstream in lower reaches (S5 and S7) of the Crowsnest River (Watt et al. 2021). A detailed description of geology, land use and landscape disturbance in the Crowsnest River basin are presented in Watt et al. (2021). Hydrometric and sediment sampling programs were conducted from May 22 to August 2, 2019, to measure instantaneous discharge, VC, and EPSD of suspended solids during three high discharge events at four study sites in the Crowsnest River (Fig. 1). Event 1 occurred during the late spring freshet, while events 2 and 3 were generated by rainfall. Both the EPSD and VC of suspended solids (< 500 µm) were measured in the centroid of flow with a LISST-200x (Sequoia Scientific, Bellevue, WA, USA) at intervals of approximately 3 to 4 days. The LISST-200 × operates on a laser diffraction principle that provides lognormal particle size distributions over 36 size bins that range from 1 to 500 μm (SEQUOIA Scientific Inc. 2018). Measurements of the EPSD and VC of SPM were made at each of the four sites for a period of 3 min, in which the LISST-200 × was configured to sample every second thus producing > 100 measurements for each deployment. Total suspended solids (TSS) was measured following the Standard Methods Procedure (APHA 1995), and the suspended sediment load was calculated according to the discharge-weighted TSS method (Nava et al. 2019). In this study, because in situ sediment concentration was measured through laser diffraction, values of TSS were only used to calculate sediment load and specific sediment yield. Instantaneous discharge at each site was either measured with a Swoffer current velocity meter (Model 2100) using the area-velocity method or obtained from gauging stations deployed by the Southern Rockies Watershed Project (SRWP; Silins, unpublished data). A comparison of flow measurements immediately downstream of S7 at Environment Canada Gauging Station (05AA008@Frank) and at Site 7 for the study period is presented in Fig. 2. A calibrated flow model (MOBED) (Krishnappan 1981) was used to provide estimates of bed shear stress in the Crowsnest River. MOBED is an unsteady and mobile boundary one-dimensional river flow model (Krishnappan 1981). Input data to MOBED consisted of the cross-sectional geometry of each transect (measured every ~ 500 m along the 10 km reach), initial bed and water surface elevation, boundary conditions at the first upstream and last downstream transects, and bed roughness parameters (Stone et al. 2021).

### Analysis of particle populations

**Fig. 3:**
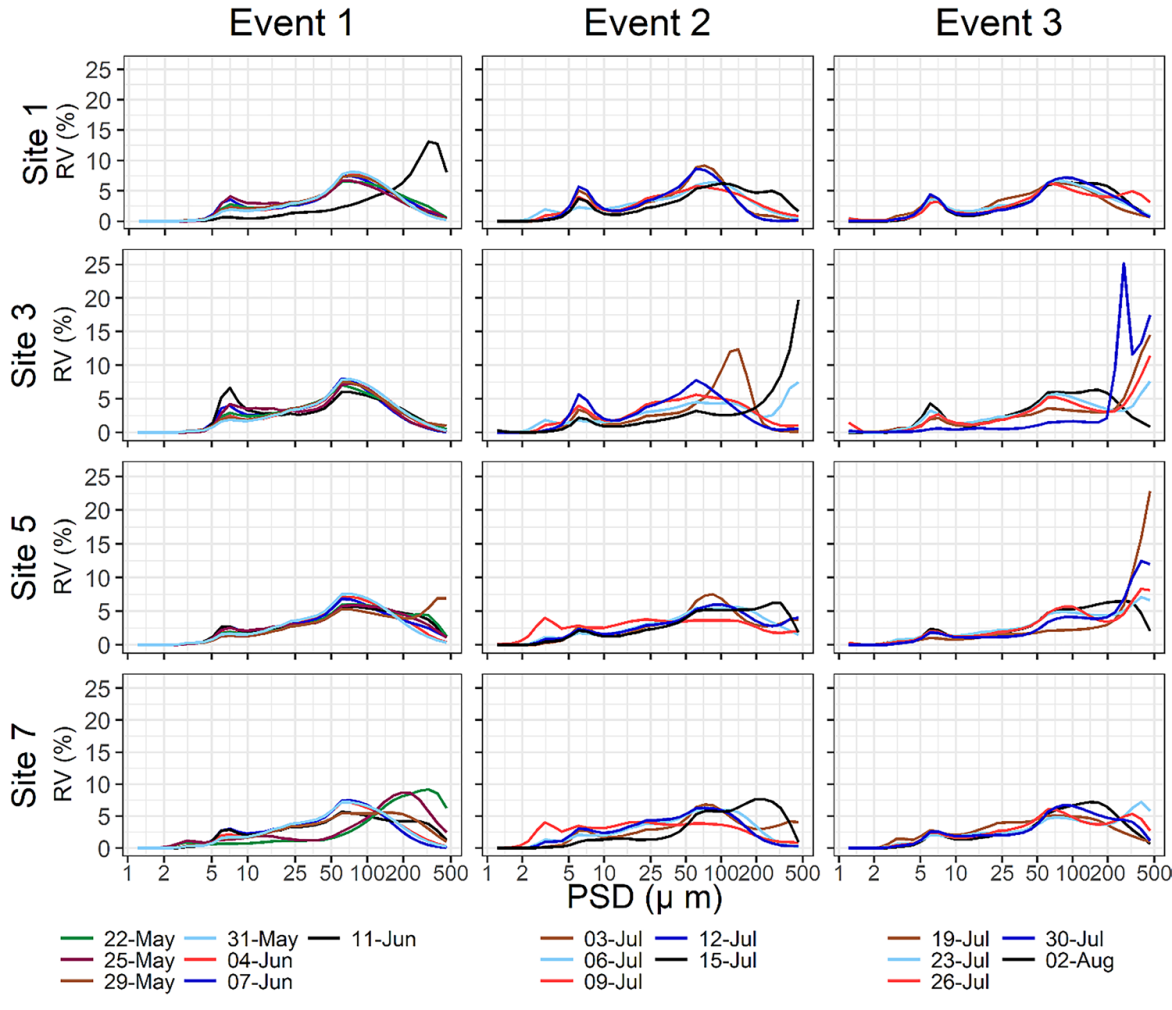
Temporal variability in the EPSDs of suspended solids in the Crowsnest River during three high discharge events

Multimodality of suspended solids PSDs and their VC were evaluated using the conceptual flocculation scheme proposed by Lee et al. (2012). Accordingly, LISST-200 × data were separated into five relevant particle size fractions: (1) primary particles (1.0 ≤ *D* < 4.7 μm), (2) flocculi (4.7 ≤ *D* < 24.6 μm), (3) microflocs (24.6 ≤ *D* < 212.0 μm), and (4) macroflocs (212 ≤ *D* ≤ 500 μm). The grouping in such size classes is performed based upon the in situ observed EPSD of SPM. Hence, if silt and sand-sized individual grains > 4.7 μm were occurring in suspension, these particles would have been classified and included in the corresponding flocculated size class. However, beyond the observations performed in our discussions (Sect. 3.1), photomicrographs taken throughout the study period (Maltauro, unpublished data) demonstrate that individual particles > 4.7 μm are seldom transported in suspension at the study sites. Although the precise definition of thresholds for size classes can be rather arbitrary (Mikkelsen et al. 2006), our thresholds were defined according to the modal peaks observed in the EPSD data (Fig. 3). Spatial variability between upstream and downstream reaches was assessed with the Wilcoxon rank sum test (Kassambara 2020), while temporal variability between the three events was assessed using the Kruskal–Wallis and post hoc pairwise Dunn’s test with Benjamini-Hochberg (BH) false discovery rate correction for multiple comparisons (Kassambara 2021). All plots were created using ggplot2 R package (Wickham 2016). Principal component analysis (PCA) was performed for data reduction, to identify key controls on the variance in the EPSDs. All statistical analysis were performed using R Statistical Software (R Core Team 2022) through RSudio Integrated Development Environment (R Studio Team 2022).

## Results and discussion

### Shear dependent flocculation and multimodality in size distributions

**Fig. 4:**
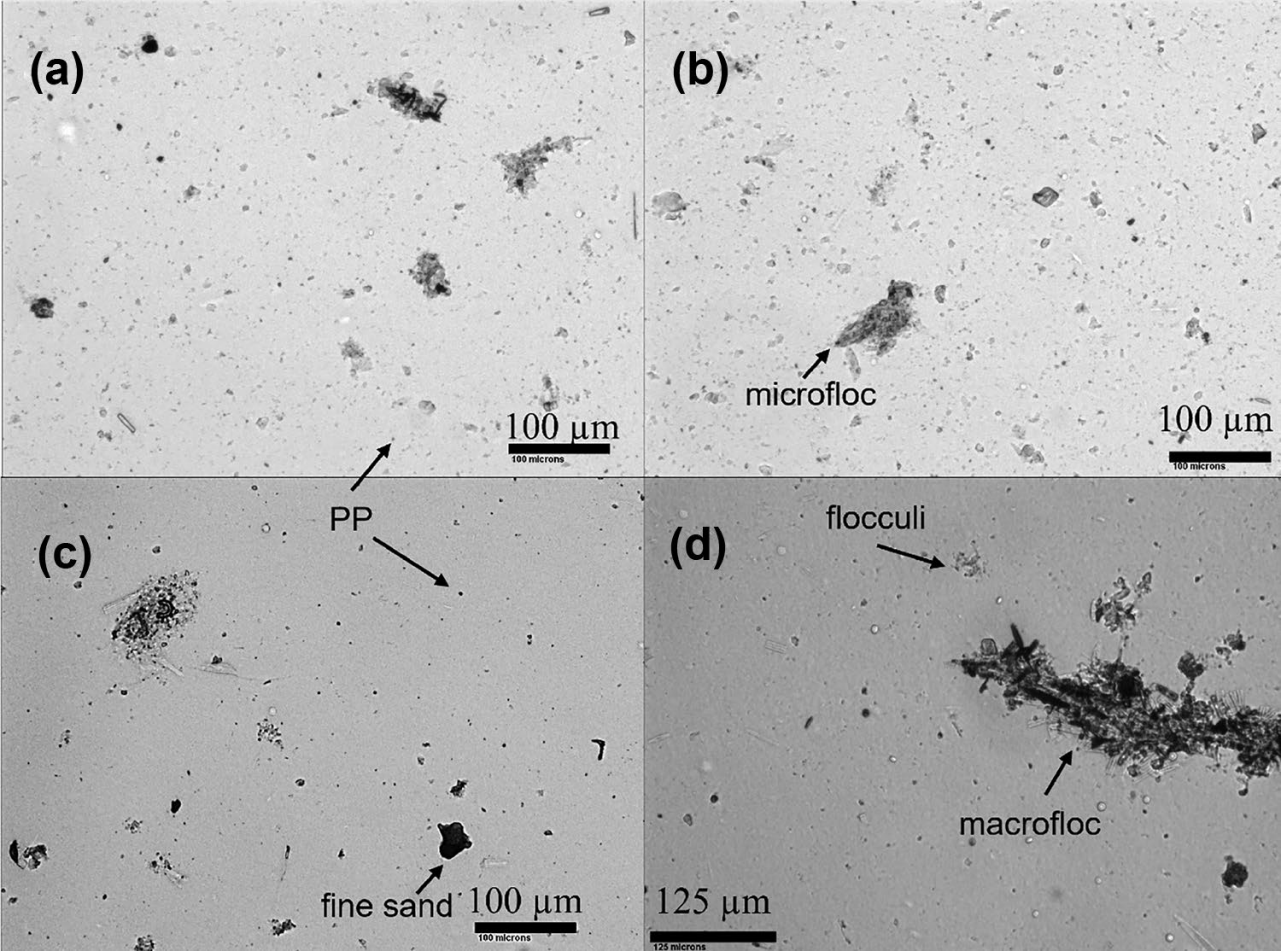
Photomicrographs of primary particles and floc classes in the Crowsnest River: **A** Site 5 on July 9; **B** site 7 on July 9; **C** S5 on May 29; and **D** Site 7 on July 30

Spatial and temporal variability in the EPSD of SPM for three high flow events in the Crowsnest River are presented in Fig. 3. River discharge, bed shear stress, VC, and specific sediment yield for each discharge event are summarized in Table 1. The EPSDs of SPM in river systems under varying flow conditions can be highly variable in time at high resolutions (Williams et al. 2007), which is in agreement with data observed in the Crowsnest River. Here, as per other studies (Mikkelsen et al. 2006; Williams et al. 2007; Lee et al. 2012), the EPSDs are presented as averages of the > 100 measurements taken during ~ 3 min at each site. Outlier values were not discarded in order to account for the natural variability in EPSDs. The relative volumes of these averaged EPSDs can be observed in Fig. 3. Relative standard deviation (RSD) was calculated for each averaged EPSD pertaining to each size bin, and the overall average RSD ($\mathrm{average\; RSD}= \sqrt{\left({\mathrm{RSD}}_{1}^{2}+{\mathrm{RSD}}_{2}^{2}+\dots +{\mathrm{RSD}}_{\mathrm{k}}^{2}\right)/\mathrm{k}}$, where k represents the 36 size bins for each deployment) for all deployments (*k* = 2448) was ~ 245%. EPSDs were multimodal, commonly consisting of two modal peaks (Fig. 3). The first modal peak was consistently observed between 5 and 10 µm, while the second modal peak occurred between 50 and 100 µm. In some cases, there were shifts to larger size classes during lower discharge conditions for events 2 and 3. The first modal peak represents flocculi size fractions, which are the building blocks of coarser flocs (Stone and Krishnappan 2003; Mikkelsen et al. 2006). Representative images of various particle size fractions (primary particles, flocculi, microflocs and macroflocs) are shown in photomicrographs of suspended solids from the Crowsnest River (Fig. 4).

**Table 1:** Variability in river discharge, shear stress, volume concentration, and specific suspended sediment yield for three discharge events in the Crowsnest River

	**Event 1**				**Event 2**				**Event 3**			
	**S1**	**S3**	**S5**	**S7**	**S1**	**S3**	**S5**	**S7**	**S1**	**S3**	**S5**	**S7**
**Initial Q (m**^**3**^** s**^**−1**^**)**	3.8	4.2	5.2	7.4	4.5	4.7	5.2	5.8	4.1	4.2	4.6	5.5
**Peak Q** **(m**^**3**^** s**^**−1**^**)**	8.7	9.4	11.2	13.3	5.5	5.8	6.8	8.7	4.4	4.5	4.9	6.5
**Average Q (m**^**3**^** s**^**−1**^**)**	6.0	6.5	8.0	10.1	4.7	4.9	5.5	6.9	3.8	4.0	4.3	4.9
^**a**^**Difference in Q** **(m**^**3**^** s**^**−1**^**)**	4.8	5.3	5.9	6.0	1.5	1.7	2.2	3.1	1.1	1.1	1.3	2.9
**Initial τ (Pa)**	26.2	22.2	16.9	22.6	28.7	24.3	18.4	23.8	27.1	23.1	17.7	23.1
**Peak τ (Pa)**	42.2	35.6	23.9	33.3	33.7	29.6	24.1	33.7	28.2	23.9	20.7	27.3
**Average τ (Pa)**	33.6	28.3	20.5	27.1	29.7	25.4	19.6	25.9	26.2	22.3	17.9	23.2
^**a**^**Difference in τ (Pa)**	16.0	13.4	7.0	10.7	6.9	6.8	6.5	11.2	3.8	3.1	4.5	6.5
**Initial VC (µL L**^**−1**^**)**	20.5	15.9	19.7	25	6.8	9.3	10.7	10.7	8.1	12.5	25.5	10.5
**Peak VC (µL L**^**−1**^**)**	46.5	41.7	51.8	42.3	14.9	14.3	24.7	22.7	8.6	34.2	25.5	13.3
**Average VC** **(µL L**^**−1**^**)**	26.4	22.7	30.6	25.8	8.9	9.9	15.0	12.4	8.1	14.6	15.5	11.0
^**a**^**Difference in VC** **(µL L**^**−1**^**)**	36.2	30.9	34.7	28.4	9.2	8.8	14.0	18.1	1.2	26.7	14.5	3.9
**Sediment load (t)**	69.1	92.6	113.1	109.5	42.1	90.0	63.7	47.2	22.1	13.3	21.8	20.0
**Specific sed. yield (kg km**^**−2**^**)**				271.64				117.01				49.53

^a^Difference between maximum and minimum observation

**Fig. 5:**
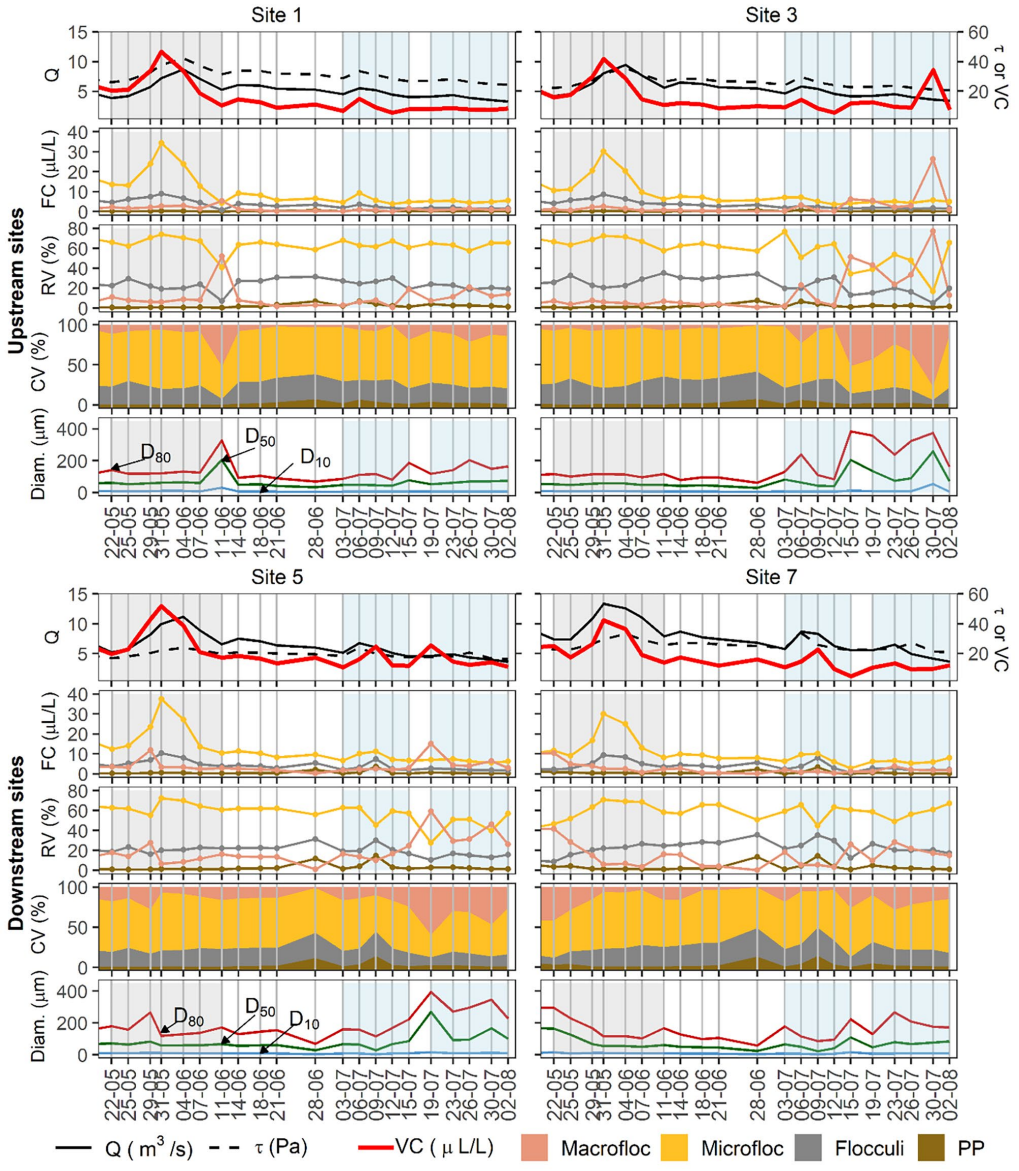
Temporal variation of measured discharge (*Q*), modelled bed shear stress (*τ*), and VC of SPM; floc absolute concentration (FC) of individual size classes; relative volume (RV) of size fractions; cumulative volumes (CV) of size fractions (where primary particles (PP) is the RV of PP, Flocculi equals the RV of PP + Flocculi, and so on; particle diameters D_10_, D_50_, and D_80_. Grey shaded areas correspond to event 1, and blue shaded areas correspond to events 2 and 3

To assess flocculation dynamics in the Crowsnest River, hydraulic (discharge and bed shear stress) and SPM (VC, particle diameters and size classes) variables were plotted as a time series for each site (Fig. 5). Differences in discharge and bed shear stress were the highest at all sites during event 1, except at site 7, at which the difference in shear stress was the highest during event 2 (Table 1). Increases in VC were generally well aligned with increases in discharge and shear stress in events 1 and 2, but responses of VC to discharge in event 3 varied at all sites (Fig. 5). Absolute (FC), relative (RV), and cumulative (CV) concentrations of microflocs were consistently predominant in event 1, but those were surpassed by macrofloc concentrations for some measurements of events 2 and 3 (Fig. 5). The flocculi class did not exceed either the micro or the macrofloc fractions during the three high discharge events, but this size class was consistently important during all events (Fig. 5). Particle diameter data suggest that particles mainly increased in size in event 3, but coarser particles were also observed in events 1 and 2 (Fig. 5). Particle diameter D_10_ was nearly constant through all events, demonstrating that finer particles are not well represented in such distributions.

Varying shear stress can influence EPSDs by altering flocculation or particle breakage, or by promoting SPM resuspension or deposition (Petticrew 2005; Lee et al. 2012). Field and laboratory studies have observed that increases in shear stress can increase particle interaction, thereby stimulating the occurrence of flocculation and the development of coarser flocs through shear-dependent flocculation (Stone and Krishnappan 2003; Mikkelsen et al. 2006; Lee et al. 2012). Flocculation occurring at higher shear stress was observed across the three study events. Increasing shear promoted the aggregation of the flocculi class into microflocs in event 1 at sites 1 and 3 between May 25 and 31. Increasing shear stress also led to the flocculation of microflocs into macroflocs during event 1 at site 5 (May 23) (Fig. 5). Shear-dependent flocculation was also observed in event 2 (site 3 – July 6) and event 3 (site 7 – July 23), when increasing shear resulted in the flocculation of microflocs into macroflocs (Fig. 5). Further, flocculation was also observed in periods when shear stress was relatively lower, such as in event 1 (site 1 – June 11, and site 7 – May 22), in event 2 (all sites – July 3), and event 3 (site 1 – July 26, site 3 and site 5 – July 19, and July 30) (Fig. 5).

Bed shear stress has a dual effect in the process of flocculation. While increasing shear stress can promote particle interactions and benefit flocculation, high shear stress can lead to interactions that are too strong for the floc structure, resulting in floc breakage (Stone and Krishnappan 2003; Petticrew 2005). Floc breakage results in decreased relative volumes of coarser flocs and increased relative volumes of smaller flocs (Lee et al. 2012). Floc breakage was consistently observed across all events. In event 1, breakage of macroflocs into microflocs were observed at site 5 (May 31), and at site 7 (May 25–May 31). In event 2, breakage of macroflocs into microflocs was observed at site 7 (July 6) (Fig. 5). In event 3, floc breakage from macroflocs into microflocs occurred when shear stress peaked at sites 3 and 5 (July 23), and at site 7 (July 23 forwards) (Fig. 5).

Decreasing volumes of coarser particles aligned with increasing volumes of finer particles were also observed under low shear stress conditions. This relationship was observed between the RV of microfloc and flocculi at event 1 at site 3 (June 11) and between the RV of macrofloc and microfloc at the end of event 3 at sites 3 and 5 (August 2) (Fig. 5). While particle deposition could have resulted in such observations (Mikkelsen et al. 2006), deposition requires bed shear stress to become lower than the SPM critical shear stress for deposition. However, previous studies have shown that this condition hardly ever occurs in the Crowsnest River (Stone et al. 2011, 2021), especially under high-discharge conditions, which is the case of our study. SPM ingress in the gravel bed could still have occurred (Glasbergen et al. 2015), and flume experiments have shown that ingress can be size-selective, thereby affecting the EPSD of SPM (Koiter et al. 2015). However, more studies are required to better understand the role of ingress on EPSDs of SPM and on the multimodality of these size distributions. Therefore, the observed decrease in particle size under lower shear stress conditions suggests the breakage of more loosely-bound coarser particles. While increasing shear might promote particle interaction and increase floc size, it is possible that once shear stress declines, flocs might decrease in size as well due to the lack of hydraulic forces promoting their size maintenance.

Changes in EPSDs can also result from the resuspension of previously deposited particles, and from particle mobilization from hillslopes and channel banks (Walling et al. 2000; Lee et al. 2012). Although high shear stresses can resuspend deposited sediment and lead to increases in the concentration of SPM (Mikkelsen et al. 2006), particle resuspension often increases the relative volumes of coarser particles in suspension (Lee et al. 2012). In this study however, such episodes were observed through relative and cumulative peaks in finer particle sizes. These peaks were observed on July 6 (sites 1 and 3), on July 9 (sites 5 and 7), and on June 28 (at all sites, but in a period outside the range of our assessed events) (Fig. 5). Further, the lower range of discharges registered in 2019 (exceedance probability of 90%), suggest that particle resuspension was likely very minimal within the studied period. While particle breakage could have resulted in such peaks, the breakage of flocs into primary particles seldom occurs naturally (Ho et al. 2022). Thereby, because these observations all occurred following a series of rainfall events (Fig. 2A), the data suggest that finer materials are likely being mobilized from channel bank or hillslope erosion. However, because the Crowsnest River has observably a well-armored channel bed and highly vegetated river banks (Stone et al. 2014), such contributions of finer particles to SPM are likely derived from upper areas in the study catchment. This hypothesis is consistent with observations reported by other studies conducted in the study area that show the potential of hillslope SPM mobilization in previously burned sub-catchments of the Crowsnest River is high (Silins et al. 2009) and that SPM from upper disturbed catchments can be transported in suspension even during low flow to the mouth of the Crowsnest River Basin (Stone et al. 2014).

### Downstream variability of VC and EPSD

**Fig. 6:**
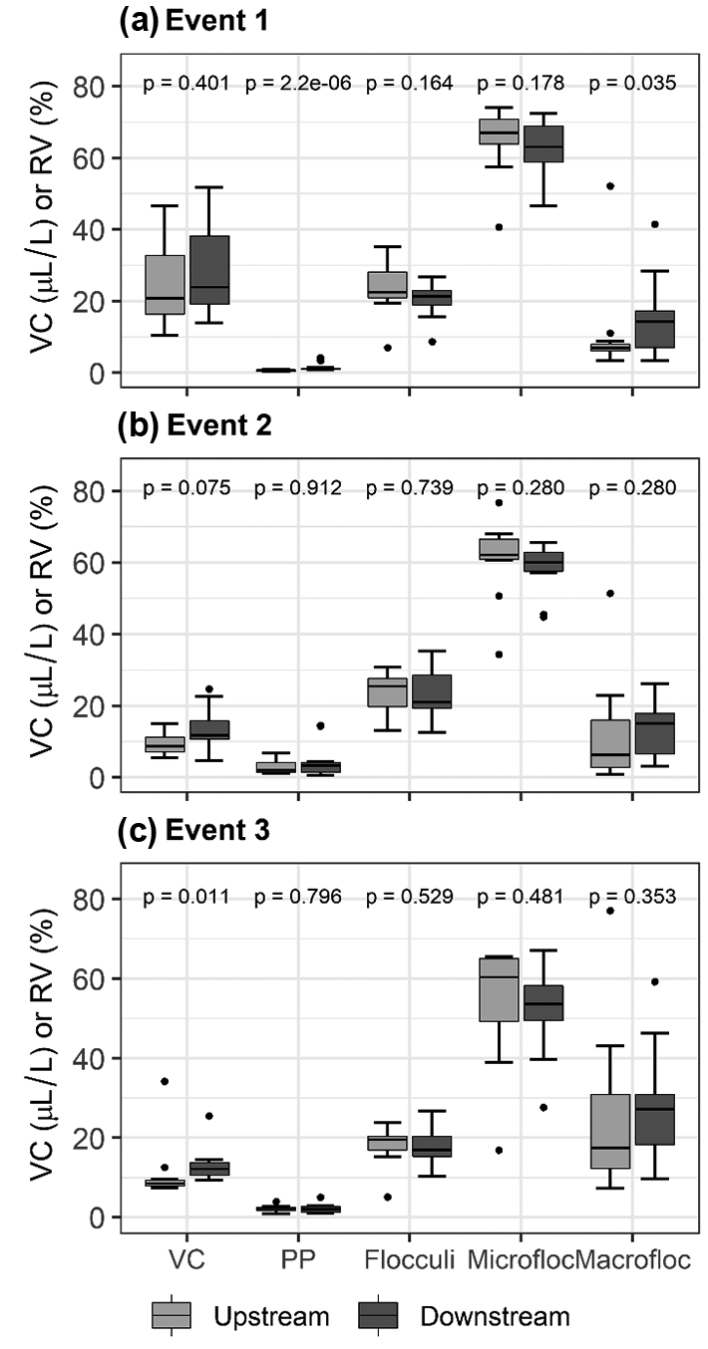
Comparison of upstream (S1 and S3) and downstream (S5 and S7) VC and floc size classes using Wilcoxon rank sum test. Median, upper, and lower quartiles; whisker indicates the range spanning 1.5 times the interquartile range. Event 1 (*n* = 14 per reach), event 2 (*n* = 10 per reach), event 3 (*n* = 10 per reach)

Study sites were grouped as “upstream” and “downstream” according to the degree of landscape disturbance to assess downstream variability in the VC and EPSD of SPM in the Crowsnest River. Upstream sites (S1 and S3) have land disturbance pressures that include industrial land clearing (such as mining), forest harvesting, urban, land clearing, and linear features (roads and ATV trails). Downstream sites (S5 and S7), in relation to upstream sites, have added natural and anthropogenic disturbances consisting of larger urban footprint, harvesting activities and wildfire (Watt et al. 2021). The median VC of SPM during all events was higher in downstream reaches compared to upstream reaches (Fig. 6). This is in agreement with previous work conducted on catchments that are tributaries to our downstream sites, which observed increased delivery of fine particulate matter from hillslope to stream networks due the intense wildfire and salvage logging activities that occurred on those catchments (Silins et al. 2009; Stone et al. 2011). The downstream increase in VC highlights the increasing downstream effects of cumulative landscape disturbances in the Crowsnest River.

Median values of the primary particle size class downstream were higher than upstream for events 1 (about 1.56 times) and 2 (about 1.62 times), but not for event 3 (about 0.87 times) (Fig. 6). These differences, however, were small, and the primary particle size class was only significantly different between upstream and downstream sites during event 1 (*p* < 0.0001) (Fig. 6). Median values of the flocculi fraction were consistently smaller downstream, and although no significant differences were observed between upstream and downstream reaches, the relative volume of this size class averaged over the three events was 1.1 times higher upstream than downstream. Even though no statistically significant differences were observed, median values of the microfloc size class over the three events were slightly higher (1.1 times) at the upstream reaches. Median values of the macrofloc fraction, on the other hand, were demonstrably higher downstream compared to upstream reaches in event 1, but less so in the other two events. The median relative volume of macroflocs over the three events was 2.1 times higher downstream than upstream.

Previous studies assessing the absolute particle size characteristics of fluvial SPM highlight that particle size selectivity can occur within channel networks. These studies attributed the increased presence of finer sediment in downstream reaches to the preferential deposition of coarser particles (Stone and Walling 1997; Walling et al. 2000). While size selectivity and preferential deposition could explain the higher occurrence of the primary particle fraction observed in the downstream reaches of the Crowsnest River, it does not explain the higher volume of macroflocs observed in the lower reach. Therefore, the downstream variations in size classes are more likely to be explained by flocculation processes. In the present study, higher relative volumes of the macrofloc class and lower relative volumes of the flocculi and microfloc classes were measured downstream compared to the upstream sites. Similar observations showing higher occurrence of macroflocs in downstream reaches have been reported elsewhere (Gibbs et al. 1989; de Boer et al. 2000; Stone et al. 2021).

### Inter-event variability of VC and EPSD

**Fig. 7:**
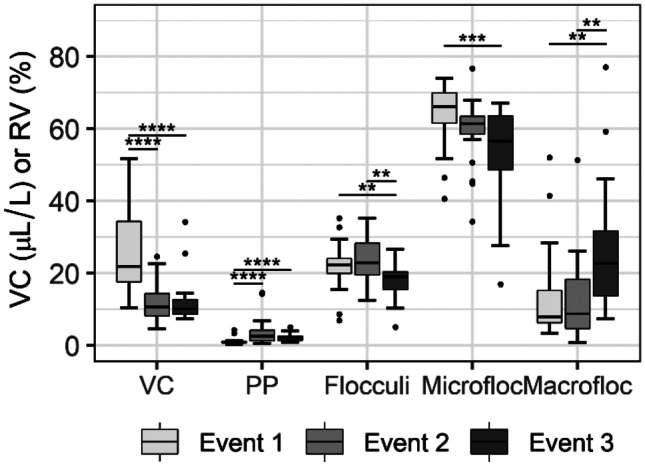
Inter-event comparison of VC and EPSD using the Kruskal–Wallis test followed by a post hoc assessment with Dunn’s test. Benjamini–Hochberg adjustments were made to *p*-values. Median, upper, and lower quartiles; whisker indicates the range spanning 1.5 times the interquartile range. Event 1 (*n* = 28), event 2 (*n* = 20), event 3 (*n* = 20). Adjusted *p*-values: * *p* < 0.05, **p < 0.01, ***p < 0.001, and ****p < 0.0001

Inter-event variability of VC and EPSD are presented in Fig. 7. Discharge measurements from the three events were all significantly different (*p* < 0.05) and bed shear stress was only significantly different between event 3 and the previous two events (*p* < 0.005). Measured discharge and modeled bed shear stress were consistently higher during snowmelt (event 1) but decreased progressively during storm events 2 and 3 (Fig. 5). The highest VC values and specific sediment yield occurred during event 1 (Table 1). While the SPM VC between events 2 and 3 was not significantly different, specific sediment yield in event 3 was consistently smaller than event 2 (Table 1; Fig. 7). These results demonstrate the importance of the snowmelt on the mobilization of higher amounts of SPM in the Crowsnest River, even during drier years (as discussed above).

Event 1 had a considerably lower volume of primary particles in suspension, indicating that this size class is more likely to be transported during rain events (Fig. 7). The two observed peaks in the primary particle size class on June 28 and July 9 (as discussed above) are believed to have occurred due to either hillslope, or tributary channel bed/bank erosion rather than from the breakage of flocculated particles. Significant differences between events in the size classes of flocculi, microfloc and macrofloc demonstrate the differences in the flocculation dynamics in the study period. While volumes of the flocculi fraction in events 1 and 2 were significantly higher than in event 3, volumes of the macrofloc class during the first two events were significantly lower than during event 3. The microfloc fraction during event 1 was significantly higher than for event 3 (Fig. 7). These comparisons suggest that flocculation during event 1 was likely limited to the microfloc size class since high bed shear stresses developed in this event limited the formation of macroflocs. In contrast, smaller volumes of flocculi and microflocs, and higher volumes of macroflocs were observed during event 3. Even though the VC during event 3 was lower than the previous events, floc size increased significantly (Fig. 7) due to lower bed shear stresses (p < 0.005) that developed during the last event. During event 2, which had comparable bed shear stress to event 1 (p > 0.05), but comparable VC of SPM to event 3 (Fig. 7), flocculation was limited to the size ranges of flocculi and microfloc fractions.

### Relationship between hydraulic properties and in situ particle size fractions

**Fig. 8:**
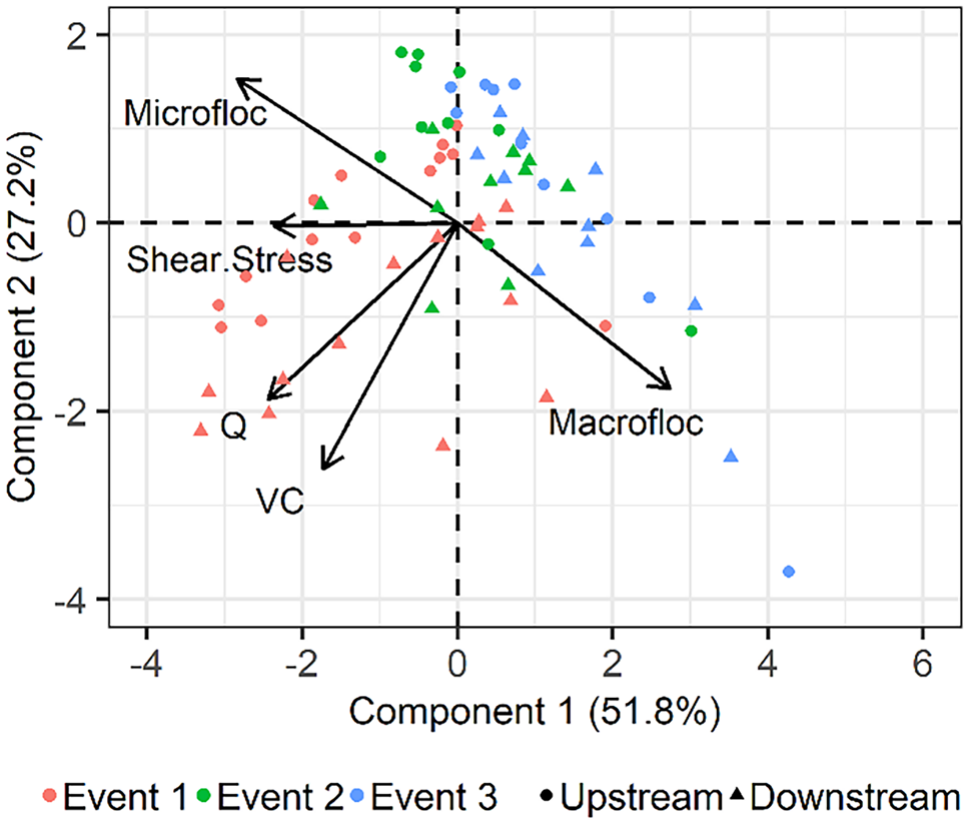
Principal component analysis (PCA) showing the first and second components, indicating relationships between hydraulic properties, VC, microfloc, and macrofloc size classes at upstream and downstream reaches during the three studied events

The relationship between hydraulic properties, VC, and microfloc and macrofloc size classes at upstream and downstream reaches during three high discharge events are summarized in a PCA biplot (Fig. 8). The first two components of the PCA explain a relatively high proportion (PC1 51.8% and PC2 27.2%) of variance in the data set. The data show that more upstream sites were correlated with the microfloc size class, while downstream sites were correlated with the macrofloc size class. Regarding the different events, event 1 was correlated with increased values of discharge, bed shear stress and VC, while events 2 and 3 were inversely correlated with these same variables. Discharge was positively correlated with increasing VC, but bed shear stress was better correlated to specific size classes. Bed shear stress was positively correlated to microflocs but negatively correlated to macroflocs. The PCA assessment shows that microfloc and macrofloc volumes are inversely correlated, showing that the relative volume of the microfloc size class decreases as these particles flocculate into macroflocs.

### Implications for modelling the flocculation process in the Crowsnest River

A modelling framework for fine sediment transport including the flocculation process, has been proposed by Stone et al. (2021) to predict the downstream transport of sediment mobilized from burned and unburned areas of the upper basin of the Crowsnest River into the Oldman Reservoir. The modelling framework includes a flocculation module, called RIVFLOC, developed by Krishnappan (1991). The RIVFLOC model uses a coagulation equation that incorporates terms that describe collision mechanisms resulting from Brownian motion, turbulent fluid shear, inertia of the sediment particles and differential settling. The cohesion that is responsible for the collided particles to bond together and form flocs was considered in terms of a cohesion-factor that was assumed to be constant through the whole spectrum of floc size distributions. The present study highlights the need to treat the cohesion factor as a variable and suggests using different values of the cohesion-factor for the different mechanisms of the formation of flocculi, microflocs, and macroflocs to allow the model to predict the multimodal distributions better. The present study also provides support for refinement of the disaggregation scheme used in the RIVFLOC model wherein the break-up of flocs due to turbulent fluctuations of the flow field was formulated using a methodology proposed by Tambo and Watanabe (1979), and using the model parameters recommended by them. The present study allows for optimizing these parameters to model the disaggregation of the macro flocs into micro flocs in the Crowsnest River.

### Study limitations

Field measurements in this study were taken at four different sites at an interval that ranged from 3 to 4 days. In agreement with our observations, previous studies assessing in situ EPSD have reported more temporal than spatial variability in the EPSD of SPM (Phillips and Walling 1999), and those using a high temporal resolution assessment have shown that EPSD can be highly variable in time (Williams et al. 2007). We did not explore the role of organic material in controlling the EPSD of the SPM in our study basin. We also did not quantify the benefits of refining the parameterization of cohesion or disaggregation within the RIVFLOC model using the new understanding assembled by the work herein and the corresponding implications for river watershed management. Similar experimental work would be needed to support the application of RIVFLOC in other river watersheds and to understand the implications of any catchment-specific refinements to the cohesion or disaggregation parameters for model accuracy and landscape decision-making.

## Conclusion

Time series of multimodal EPSDs were evaluated for three high discharge and turbulent shear events in a gravel-bed river in southern Alberta. The multimodal EPSDs which consisted of primary particles, flocculi, microflocs and macroflocs were dynamic under different conditions of turbulent shear. In agreement with the general theory of flocculation, the results of this study show that shear-dependent flocculation was the primary mechanism causing multimodal shifts in the EPSDs of SPM. At low turbulent shear stress, the EPSDs generally skewed towards a larger particle volume of micro and macro flocs suggesting an aggregate-dominant condition. However, under conditions of higher turbulent shear, EPSDs skewed toward smaller size fractions and a large volume fraction of floc building blocks were observed in a breakage-dominant condition. These observations have implications for advancing fine sediment transport models by a variable cohesion factor as a function of floc size class. Here, flocculation and particle breakage processes were assessed based on relationships between particle size and hydraulic properties. Further research is still required to better understand physical, chemical, and biological processes that govern flocculation. In addition, further investigation is still required to better understand the role of gravel-beds and ingress mechanisms on flocculation processes.

## Data Availability

The datasets generated in the framework of this study are available from the corresponding author upon reasonable request.
